# American Dental Association

**Published:** 1874-12

**Authors:** 


### SELECTED ARTICLES.

### ARTICLE IV.

*American Dental Association.*—CONTINUED.

SECOND DAY.—*Morning Session.*

The subject of "Dental Pathology" was still under consideration.

Prof. Taft. Enamel may be diseased accidentally or hereditarily. There is great variety in enamel: some defects are transmitted; others are owing to an interruption of the nutritive process. It is often defective through absence of perfect crystallization; there are defective tracts, —atrophied portions, owing to disease occurring for a time. This is shown by spots of a different color, or grooves or pits, at the points which are forming at the time of the affection. Fissures present openings, especially in molars and on the lingual surfaces of the front teeth. There are defective tracts, beginning internally and extending to the periphery,—or part way. These are different from pits, and are often found on the cusps. How do they occur? By their situation it is rendered impossible that anything should be retained which should cause decay by decomposition. Sometimes there is no organization; the materials are simply thrown together. The fibrillæ sometimes extend into and through the enamel. In one specimen he had examined, the largest number he had ever seen seemed to pass through. We should find out how these phases will modify treatment. We should understand at a view the structure of the teeth. Disease leaves its tracks upon all the tissues, and observe whether they are in their original power. The dentine is not open to observation, but still we may be able to discover something. We cannot understand a portion of a science without covering the whole ground. Let us criticise as sharply as possible, and see how comes every defect; and endeavor if possible to redeem even those that are hereditary. We must stimulate one another to investigate at home. As regards the parasitic theory, he has not worked himself up to a great amount of interest in it. Because there are living animals in a dead carcass, it does not follow that they were the cause of the death. They play no important part in the decomposition of dentine. Worms and bugs in dead wood are not the cause of its death, but the result. Parasites have no material part in causing decay. The mode of penetration of caries proves nothing as to the agency of these animalculæ. How does it begin? Sometimes it is superficial and is arrested, sometimes largely beneath the enamel; sometimes it penetrates like a shot to the pulp-chamber. These varieties are easily accounted for. The structure between the enamel and dentine varies exceedingly in thickness and character. If it is defective, the decay will extend largely beneath the enamel. But if it is good, the decay will be penetrating. It operates in the direction most easily penetrated. Sometimes the decay-producing agent is concentrated, and most active at one point.

Dr. Spelman. Recent histologists discard the theory of parasites, and substitute that of vegetable or fungous growths. Parasites are found in every species of decay. When decomposition takes place, the open rods are exposed to acids and the leptothrix penetrate; as it progresses, they enter the tubuli and proceed till the pulp is reached. The most natural cause of caries is a growth of vegetable fungus, throwing out spores which articulate with the parent fungus. They develop best in an acid condition. The mouths of the tubuli are enlarged.

Dr. Watt. In regard to the "tooth-edge" business, we overlook the fact that it is not always traumatic; sometimes it exists where there are no natural teeth, and even seems to be located in artificial teeth. This is the same phenomenon as apparent pain in limbs which have been lost. An alkaline wash relieves temporarily. Microscopical examinations do not always reveal the "bugs." White decay is the most penetrating, and it is found in alkaline mouths. Alkalinity in mouths is due to the presence of ammonia; this, in contact with air, is converted into nitric acid, which attacks everything; it has a stronger affinity for lime salts than for anything else. Ammonia is produced in the breath; it produces spongy gums and soft and porous salivary calculus. Carbonic acid keeps the lime in solution; heat drives off the carbonic acid, and the lime is precipitated. He had been represented as teaching that caries was wholly chemical, in spite of the care he had taken to say that vitality always modifies chemical action. There are four varieties of decay, and many crosses.

Dr. Morgan. Is impressed with the importance of the idea that disease leaves its impress upon the tissues. Whatever interferes with nutrition impairs the dentine. This shows the importance of the utmost care in respect to the temporary teeth. If they are interfered with so as to arrest the development of the permanent, lasting injury is done.

Prof. Judd. We need something more than we had fifteen or twenty years ago to explain carious action. He has never felt satisfied with any of these theories, but had been in hopes of a better. With regard to the vital theory, one fact is to be considered. Extract a tooth, and reinsert its crown in the mouth, and a carious cavity will occur. He does not deny that the vital force exerts some influence, but in view of the above fact it cannot be the governing principle. The acids of the mouth have a great influence, but carious cavities are not produced by immersing teeth in acids. There is a general effect produced, but no cavities. Is satisfied that *one* agent has been discovered,—the parasite. There are few microscopists in this country capable of examining this subject; but we have the evidence of the best microscopists that parasitic germs exist. How do they act? I cannot tell. Some claim that there is a distention of the dentinal tubuli; this may be; or it may be that they exercise an immediate influence on the soft tissues. Again, it has been surmised that the parasites increase the acidity, and this is a very probable theory.

Dr. Spelman. The acid theory is an accepted one, but does not account for decay. Parasites assist to dilute the fluids, and prevent destruction. Fungous growths prevent motion and dilution. The parasitic theory is being discarded.

Prof. McQuillen was surprised that any one should deny the existence of parasites in carious cavities. He had not only seen them, but watched their movements over and again for hours. As to their causing decay, that is another question. A gentleman claimed last year in the Southern Dental Association to have seen parasites with formidable boring apparatus, by which they worked their way through the enamel and dentine, but we are yet in doubt as to the nature and character of these parasites,—whether they are vegetable or animal. The majority of investigators claim that they are vegetable. Whatever the other causes at work may be, we cannot deny the action of acids in the production of decay. When it is asserted that vitality controls chemical action, it seems to me that those who assert it do not understand what is meant by vitality, or the correlation of chemical and vital force. Instead of chemical affinities being controlled by vitality, there is no vital action possible without the incessant and complicated actions of chemical affinities; all the molecular changes of composition and decomposition occurring in the living body —nutrition secretion, and motion—depend on chemical actions. Vitality does not prevent the destructive action of an external chemical agent, it only repairs its ravages. Dip a dead man's finger in nitric, muriatic, or sulphuric acid, and the cuticle will be destroyed never to be replaced; subject the finger of a live man to the same treatment, and the vital action will restore the lost tissue.

Dr. Atkinson. Vitality in its fullest extent includes all other kinds of action. Chemical action is a department of vital action. It is pitiable that men will have the hardihood to open their mouths before this body and not know the thinnest skim-milk significance of the terms they use. One speaker says that parasites have nothing to do with it, and then gives us the description of a parasite. Enamel is one of the differential tissues of the body. The first cell must have been calcified in order to constitute it enamel at all. We cannot transpose the natural serial order. What is the use of talking about dissolving enamel that never existed? When vitality is least, resistance is greatest; and where it is highest there are more doors to let in Peter and Paul and kick up a bobbery in the household. If the elements are perfectly saturated, they are married for life; there is no free love; and if they are not satisfied (as we would say of men and women,) you had better look out how you let visitors into the family. You can never get enamel tissue till you have a magma to set it on by the proliferation of the stellate cells that are the base of the rods of enamel. We are mere beginners, and must speak alphabetically. Teeth with more enamel material than is necessary are found to have little pearls at the bifurcation of the roots. People are cultured that are developed in society, and the rule is as good in molecules as in men. When the bonds of affinity are satisfied, we have a permanent tissue. Enamel is an example of crystalline structure; dentine is a compromise. Calling teeth dead is nonsense; they are not dead so long as they hold connection with the system. In teeth out of the mouth, black decay cannot be produced except through a long series of alternating experiments. All decay is affected by solution. Enamel grows upon a basis of dentine, and when full-formed it is next to impossible to affect it in the mouth except by abrasion, because the other tissues will feel the attack so much as to notify the owner.

Prof. Taft. In decay of the teeth, the best organic structure possesses the highest manifestation of vital principle, and in consequence best resists decay. If the life principle is removed, the tissue disintegrates and falls to pieces. As the life-force is toned up and vigorous, it will resist.

A committee, consisting of Drs. Bogue, Taft and Shepard, was appointed to prepare resolutions on the death of Prof. Hitchcock.

The report of the Committee on Dental Chemistry was called for, and was read by Prof. H. A. Smith.

The report stated that the year had not been prolific in investigation in this department, but such investigation requires special training, severe study, means, and leisure, and we congratulate ourselves that so many practical applications of discoveries have been made. Apparently valueless ones should not be judged hastily; they may be of great importance. Practical applications are not generally made by discoverers; witness chloroform, discovered by Liebig in 1834, and applied by Simpson long after, and nitrous oxide, discovered by Davy and applied by Wells to anæsthesia.

The report then alluded to the translation from the French by Prof. Judd of a paper on Dental Tartar, by Dr. Vergue, and commended it as valuable to the student.

Prof. Chase had once read a paper before this body, claiming that human saliva was acid or neutral; but this experimenter stated that it was alkaline, considering that the acidity was the result of admixture with other substances in the mouth. Tartar was then described; its different colors attributed to different agents; and an analysis of it given, from which it appeared that the phosphate of lime is found in much greater quantity in the tartar of the incisor teeth, and the phosphate of iron predominates in the molar. The author concludes that tartar is produced by a change in the gingivo-dental deposit, by which it is decomposed; and that it is formed uniformly upon each tooth, but mechanically collects at two points.

The report then referred to a series of experiments by Dr. G. V. Black, of Illinois, as to the causes of the various colors observed in decay. The fact is noted that all decomposing animal matter is darkened in color. This was stated to be owing to sulphuretted hydrogen, which precipitates any metals present, forming sulphurets, some of which are black and insoluble. That the same causes produce the colors in decayed teeth is highly probable. The decomposing pulp blocks the tubuli, and the sulphurets turn in dark. The sulphurets of iron may be formed from the blood. The experiments to sustain this theory were, in brief, a solution of a tooth in hydrochloric acid, forming a colorless solution ; sulphuretted hydrogen gas was then passed through the solution, which changed it to a yellowish color: and the tooth itself (which was immersed) was also changed to a color corresponding to the light yellow of decay. The solution being neutralized by soda, the color grew dark, and finally black, as the reaction became alkaline. By varying these experiments all the colors of decay were produced.

Some further experiments by Dr. Black were described, showing the effects of acids in motion upon the teeth. An apparatus was devised, causing an acid solution to move constantly; two sound teeth placed obliquely in the current were cut gradually away, beginning at the cusps ; a groove was also cut between the teeth. On the third day this had extended into the dentine. On the sixth day the crowns were going to pieces. It was noticeable that the cutting was least upon the surface placed fairly against the current, and most where it broke around the tooth. A tooth immersed in the same solution, but without motion, showed only the usual slight softening. Dr. Black is convinced that this will prove to be the true explanation of decay.

The report then adverted to the action of the coloring matter in vulcanite upon the mouth, and its effects upon the health. There are two opinions; the minority think it highly injurious ; while the majority claim that it is inert. Vermilion is six parts of mercury to one of sulphur, and forms thirty-six per cent. of the whole mass of rubber. It is insoluble except in nitro-muriatic acid. The tendency to

"flower," or appear in minute globules, may, by the globules being liberated by friction on the plate, and the decomposing action of light, free a small quantity of mercury, which being introduced into the stomach in a finely-divided state might be acted upon by the acids there present and the active chlorides of the metal produced. Some varieties are adulterated with arsenic, etc., to which the deleterious effects may be due. In such cases, however, it should be ascertained whether mercury has not recently been taken. It may indeed stay a long time in the system, and then suddenly manifest itself after taking, for example the iodide of potassium.

The difference of opinion on this subject reflected little credit upon a body claiming to practice on scientific principles. If the compound is injurious, it is our duty to abandon it. The association should institute an investigation.

Dr. Shepard gave notice of an amendment to the constitution, giving the Executive Committee the power to change the place selected for regular meetings for extraordinary reasons. Laid over till next year. Adjourned.

The afternoon session was dispensed with, to admit of the attendance of the association *en masse* upon an excursion provided by the liberality of the dentists of Detroit. A steamer was chartered for a trip down the Detroit River to Grosse Isle, a journey of an hour or more. At the island where a retired hotel afforded a quiet and inviting retreat, a collation was served, to which ample justice was done by the party; and in a short time they were again embarked and on the "home stretch." The beautiful scenery along the bank, the enlivening strains discoursed by a fine military band, together with social intercourse, rendered the whole affair an exceedingly enjoyable and pleasant one. A landing being effected, the excursionists marched in procession directly to the hall and at once organized for the

*Evening Session.*

The subject of "Dental Chemistry" was passed, and "Therapeutics" called, but no member of the committee was present. "Dental Etiology" was then called, and a report read by Dr. M. H. Webb. We give the following synopsis:

Etiology is the science of causes; dental etiology, the causes of processes destructive to the dental tissues. During the formative process, there may be deficient nutrition, or there may be an interruption of nutrition by reason of disease, either of which results in insufficient density or permanent defects of structure. Teeth bear the impress of hereditary disease. The etiology of dental caries embraces imperfect tooth-structure; insufficient calcification of the enamel prisms, incomplete coalescence of the prisms, too great interglobular spaces; and fissures in bicuspids and molars. Certain conditions favor the production of acids as well as of parasites, which Lieber and Rottenstein regard as having much to do with caries. Wedl says it is at least accelerated by them. The former experimenters obtained with dilute acids results identical with those of Westcott, Allport, and others, but did not succeed in imitating caries. Lactic acid is most abundant in the mouth, though various other acids are found. They need not be strong to separate the carbonic acid from the lime. Enamel is normally translucent, dentine opaque; but when attacked by acids, the former becomes opaque while the latter becomes translucent. After the dissolution of enamel, the leptothrix penetrates deeply into the dentine and tubuli, which become enlarged, even in superficial caries; beds of leptothrix are found in uncleansed places. Acids and leptothrix may be reasonably inferred to penetrate the dentine and accelerate disintegration. If caries be superficial, it may be arrested by the removal of the decayed portions, but if the dentine is involved, it cannot be determined when the leptothrix are eradicated,—and if they are not, caries will probably recur; acids favor the proliferation of the parasites. When decomposed material is allowed to remain at the bottom of cavities, the fungi perish or may be destroyed by carbolic acid. When dentine is attacked it is best to excavate and fill, rather than cut away and obliterate the decay Calcareous deposits sometimes fill the tubuli, known as secondary dentine; Magitot, as well as Lieber and Rottenstein, believed them to spring from the pulp; Mr. Tomes considers it probable that this is deficient in vitality, and this view is favored by its want of sensitiveness. Acids will also attack this, notwithstanding the barriers erected by nature. Polished surfaces are merely the mechanical result of mastication. Salivary calculus causes absorption of the alveoli. The salts of the saliva are precipitated and deposited upon the teeth, and leptothrix, leucocytes, etc., are embodied in masses of this substance. Where it exists, decay is least active, and vice versa. The sublingual secretions are alkaline, while that of the parotid is acid; when it becomes alkaline, the salts are precipitated. When the secretions are decidedly alkaline, the salts are dissolved instead of precipitated, and caries proceeds with vigor; the pulp is reached, and the whole train of dental diseases—odontalgia, abscess, and sometimes consequent necrosis—ensues.

After the reading of this paper, the subject was deferred till the remaining reports should be read. "Operative Dentistry" was then called for, and a report read by Dr. Shepard.

The report mentioned as among the improvements in this department which has made rapid strides during a year or two past, machinery for operations upon the teeth. It has met a universal want, and though when used without judgment causes the loss of valuable tooth tissue, is yet indispensable. Two things are yet wanting to perfect it, an automatic motor, and a means of stopping and starting the bur suddenly in the mouth. The first is necessasy because no nervous force can be spared to drive the machine. Electricity has been tried, and cannot be regarded as a success. Steam has objections, but they may be overcome. The simple idea of its use will tend to prevent its general employment. Several water-motors have been invented, two of which were mentioned as being applicable; also a spring-motor, which is contained in a box a foot square, and can be used anywhere.

The report then alluded to the fact that a few years ago the tendency was to the use of heavy mallets, and that more recently there has been a tendency in the opposite direction, which by some has been carried to the extent of simply rubbing down the foil with the slightest pressure. Ivory points had been recommended, but had not proved a success. Very hard steel points were also recommended, and were claimed to be less dangerous to fragile walls; also a plugger, with a point consisting of a serrated wheel, designed for use after some progress has been made in the filling; this is agreeable to the tired patient and to the tooth sore from malleting. Fisk's saliva ejector was spoken of and recommended. The use of platina foil was mentioned, and recommended for restoring contour, the color being more agreeable than gold. A new preparation of amalgam in pellets was also mentioned favorably.

The report then adverted to the discussion as to the comparative merits of cohesive and non-cohesive foil, and referred to the experiments made by Fletcher, of England. Similar ones made in this country have proved that tight fillings can be made with cohesive gold; poor ones are made with it as well as with all other materials. But have we not used too much cohesive gold to save the largest number of teeth for the largest number of patients? A reaction is sure to come; many of the most active and progressive men have been seeking it out in quiet; and the foil-makers sell more of soft and less of cohesive. Rubber dam and cohesive gold are the greatest discoveries of the century in our art,—all honor to their discoverers,—no man can do first-class work without these articles. Our meetings, literatures, and clinics show how they are appreciated. Of late, however, the question, how best to serve the patient with the least expenditure of time and suffering, is forcing itself forward. The interests of the patient are paramount to those of the profession, and we sometimes lose sight of this and proceed as if he had no rights. Filling teeth is a longer and more painful operation than formerly. Is this necessary to save them? We ought to devise some way in which good, cheap dentistry may be placed within reach of the masses. Every unnecessary moment spent is a fraud upon the patient. The tendency has been to this extreme, and it is high time that some one should champion the sufferers. We have run mad upon cohesive gold, rubber dam, and the mallet, and have abused them shamefully. We are divided into three classes: the enthusiasts, trying all new things, and casting aside the old —among these are found some of the world's benefactors; the old fogies are at the other extreme; they are safe men, but of little benefit so far as advancement is concerned, and of little influence with the profession. The other class is the mean between these extremes; they investigate, await results, and form the jury whose verdict stands the test of time. The general practitioner should enroll himself among this middle class. Cohesive foil should be used only when it is necessary to have strength. Why put our patients to the expense of time and pain except in these cases? To apply the dam takes time, and is painful; when the tooth can be filled without it its use is injudicious and unkind, and keeps patients away, from their fear of it. The writer uses it less than he did five years ago. It is unnecessary in four-fifths of medium crown cavities, labial cavities in incisors, even under the gum, and in many proximal cavities Have the crown fillings of ten or twenty years ago failed. Our much vaunted progress has not been all upward. There are many teeth which a regard for the interests of the patient require to be filled with other material than gold. Tin in crown cavities not having antagonists, as well as in many proximal cavities, is recommended. It is of low conductivity, and is antiseptic. Gutta-percha is a most valuable filling for buccal cavities of molars, and for children's incisors. These two should be used instead of the ton and a half of amalgam now used yearly.

The subject being open,

Dr. Southworth said that the amalgam pellets, as prepared, are extremely dry, and difficulty will be experienced in their use by those accustomed to the ordinary article, which is about half mercury. The pellets have but two-fifths. Where the dam is difficult to apply without using wedges, use a little toilet soap upon the teeth, and have no further difficulty.

Dr. Crouse takes many exceptions to the report. It accuses operators using machinery of cutting away too much. This is not generally true; the failure is the other way, according to his observation, ever since we have had machinery. The electric machine *is* a success; has none to sell, but will stand up for it. There might be an improvement, however, in it; and one, as to stopping the bur, has already been made. Agrees with the paper as to the use of soft foil. Never used cohesive universally. If the report means by soft foil partially cohesive foil, will agree with it; this is more easily adapted than very cohesive. For contour fillings, uses Nos. 60, 120, or 20. Hand-pressure should be used more than it is, especially in chalky teeth, which must not be battered at the edges. The report advocates tin foil where there is no antagonist. It takes but little more time or expense to fill with gold; then why not use it? It will be a good thing when we come to the time when we can fill teeth rapidly and well, so as to bring dentistry within the reach of the masses. The dam causes very little pain, takes little time, and is pleasanter than napkins, and you can see your cavity and work with ease and confidence.

Dr. Morgan protests against the use of the terms soft and hard foil, as used. The softest foil is the most adhesive.

Dr. Wetherbee. The report is incomplete, in not mentioning filling roots or removing salivary calculus. He enters his caveat against the statement that labial or buccal cavities can be as well filled without as with the dam. The gum will be wounded and will bleed; the gold is liable to come in contact with it. The patient is not incommoded by thin rubber and fine twine. The majority of patients will prefer a clean piece of rubber to a napkin under the lip. In cavities under the gum an instrument is carried through the hole and below the margin of the cavity, and held by an assistant, and the twine is carried up carefully and tied. You now finish the excavation, and go on with your filling, which can be finished off without lacerating the gum. Some cavities may be filled with pellets without the dam, but they must be small to be successful. Formerly had written against the mallet, unfortunately, but tried it, and found himself not so tired, and not one patient in ten objected. He had dispensed with the light mallet, and used a lead one of five and a half ounces, and found a less number of devitalized teeth giving trouble. He was a convert to the mallet, and rejoiced that it was introduced. For root-filling it has been claimed that oxychloride and Hill's stopping are as good as gold. Few have studied the subject so carefully as to determine this; gold is superior to either under all circumstances. It may be more difficult, but the head and hands should be educated to the task. A professional man should know no defeat where it is possible for intelligence to gain the day. Gold can be carried with certainty where oxychloride, etc., cannot. The subject of salivary calculus has been neglected. We should be diligent in our search for it. It is doing serious injury, and there are no suitable instruments found among the profession for its thorough removal. We should battle early and long for the faithful removal of this substance, even to the ends of the roots of the molars. Proper instruments can be, and have been, constructed.

Dr. Thomas expressed his approbation in the main of the report. We are really in advance of the past, perhaps, in a hundred-fold ratio. If the report was analyzed, all would agree with it. Has seen the folly of a great deal of this progressing operating. Would not throw a wet blanket upon progression, but we should stop and consider what we are doing. Five years ago would have declared that any man that did not use cohesive foil was behind the age; but when he sees fillings of twenty to forty years' standing still doing well, he stops and considers whether it is justifiable to throw aside all of the old ways and accept all of the new. The middle ground would be more safe. Every one thinks the appliances he has used are the best. It is dangerous ground to denounce old things without a thorough investigation. Agrees with the paper that too much has been cut away; also with Dr. Crouse, when he says that we are at fault in not cutting away enough. We should pursue a happy medium in order to produce the best results.

An informal clinic was appointed for the next morning, with Drs. Webb, Butler, and Ambler as operators.

Adjourned.

TO BE CONTINUED.

